# ‘ThinkCancer!’: randomised feasibility trial of a novel practice-based early cancer diagnosis intervention

**DOI:** 10.3399/BJGPO.2023.0220

**Published:** 2024-09-04

**Authors:** Stefanie LJ Disbeschl, Annie K Hendry, Alun Surgey, Daniel Walker, Nia Goulden, Bethany F Anthony, Richard Neal, Nefyn H Williams, Zoë Susannah Jane Hoare, Julia Hiscock, Rhiannon Tudor (RT) Edwards, Ruth Lewis, Clare Wilkinson

**Affiliations:** 1 North Wales Centre for Primary Care Research, Bangor University, Wrexham, UK; 2 North Wales Organisation for Randomised Trials in Health and Social Care (NWORTH CTU), Bangor University, Bangor, UK; 3 Centre for Health Economics and Medicines Evaluation (CHEME), Bangor University, Bangor, UK; 4 Department of Health and Community Sciences, Exeter Collaboration for Academic Primary Care, University of Exeter, Exeter, UK; 5 Department of Primary Care and Mental Health, University of Liverpool, Liverpool, UK

**Keywords:** Think Cancer, Practice-based, General practitioners, Primary healthcare, Cancer, Randomized controlled trial, Feasibility studies, Diagnosis

## Abstract

**Background:**

UK cancer deaths remain high; primary care is key for earlier cancer diagnosis as half of avoidable delays occur here. Improvement is possible through lower referral thresholds, better guideline adherence, and better safety-netting systems. Few interventions target whole practice teams. We developed a novel whole-practice team intervention to address this.

**Aim:**

To test the feasibility and acceptability of a novel, complex behavioural intervention, ‘ThinkCancer!’, for assessment in a subsequent Phase III trial.

**Design & setting:**

Pragmatic, superiority pilot randomised controlled trial (RCT) with an embedded process evaluation and feasibility economic analysis in Welsh general practices.

**Method:**

Clinical outcome data were collected from practices (the unit of randomisation). Practice characteristics and cancer safety-netting systems were assessed. Individual practice staff completed evaluation and feedback forms and qualitative interviews. The intervention was adapted and refined.

**Results:**

Trial recruitment and workshop deliveries took place between March 2020 and May 2021. Trial progression criteria for recruitment, intervention fidelity, and routine data collection were met. Staff-level fidelity, retention, and individual level data collection processes were reviewed and amended. Interviews highlighted positive participant views on all aspects of the intervention. All practices set out to liberalise referral thresholds appropriately, implement guidelines, and address safety-netting plans in detail.

**Conclusion:**

‘ThinkCancer!’ appears feasible and acceptable. The new iteration of the workshops was completed and the Phase III trial has been funded to assess the effectiveness and cost-effectiveness of this novel professional behaviour change intervention. Delivery at scale to multiple practices will likely improve fidelity and reach.

## How this fits in

Primary care is a setting of importance in early cancer diagnosis, as most cancers are diagnosed here. Although UK primary care has a good track record internationally in this practice, there is variation and evidence of avoidable delays.

In addition to lowering GPs referral thresholds, safety-netting systems and back-office practices need to be improved. ‘ThinkCancer!’ may help; this bespoke, novel, complex behavioural intervention is aimed at the whole practice and appears feasible.

## Introduction

General practice teams play a key role in timely cancer diagnosis in the UK.^1^ Cancer survival rates are low in the UK compared to other high-income countries.^2,3^ Late diagnosis is a major contributor. Around 60% of cancers are diagnosed through primary care and almost half (49%) of avoidable delays occur within primary care.^4,5^ Patients delayed in the diagnostic pathway are likely to have a longer diagnostic interval and lower cancer survival.^5,6^ The gold standard is to refer patients with symptoms and signs with a 3% positive predictive value for cancer.^7^ However, the complexity of the task means guidelines are often unclear and strategies vary between GPs, illustrating the potential for improvement.^8–11^ Effective safety-netting systems in primary care must also be optimised to speed cancer diagnosis.^12,13^

A systemic approach to improve primary care cancer referral is recommended.^1^ Tailored multidimensional educational interventions have potential to reduce pathway delays.^14–16^ The topic remains urgent, as the COVID-19 pandemic worsened delays in cancer diagnostic pathways.^6,10,17^

‘ThinkCancer!’ is a complex rigorous behavioural intervention aimed at general practice teams, developed through the Medical Research Council Framework for complex interventions.^18^ Our research question was whether ‘ThinkCancer!’ was feasible and acceptable, and whether outcome measures could be adequately collected in the UK, using Wales as an exemplar.

## Method

This feasibility study incorporated a pragmatic, multi-site, two-armed, feasibility randomised controlled trial (RCT) with embedded process evaluation and feasibility economic analysis. The aim was to recruit 23–30 practices to establish feasibility and acceptability, and allow adaptation of the intervention. The unit of randomisation was the general practice, using a dynamic adaptive algorithm stratified by health board, allocating on a 2:1 ratio in favour of intervention.^19^ Harms were recorded prospectively. Participating practices were recruited between March 2020 and May 2021 (plus 5 month COVID pause). Workshops were delivered between December 2020 and May 2021.

**Figure 1.**
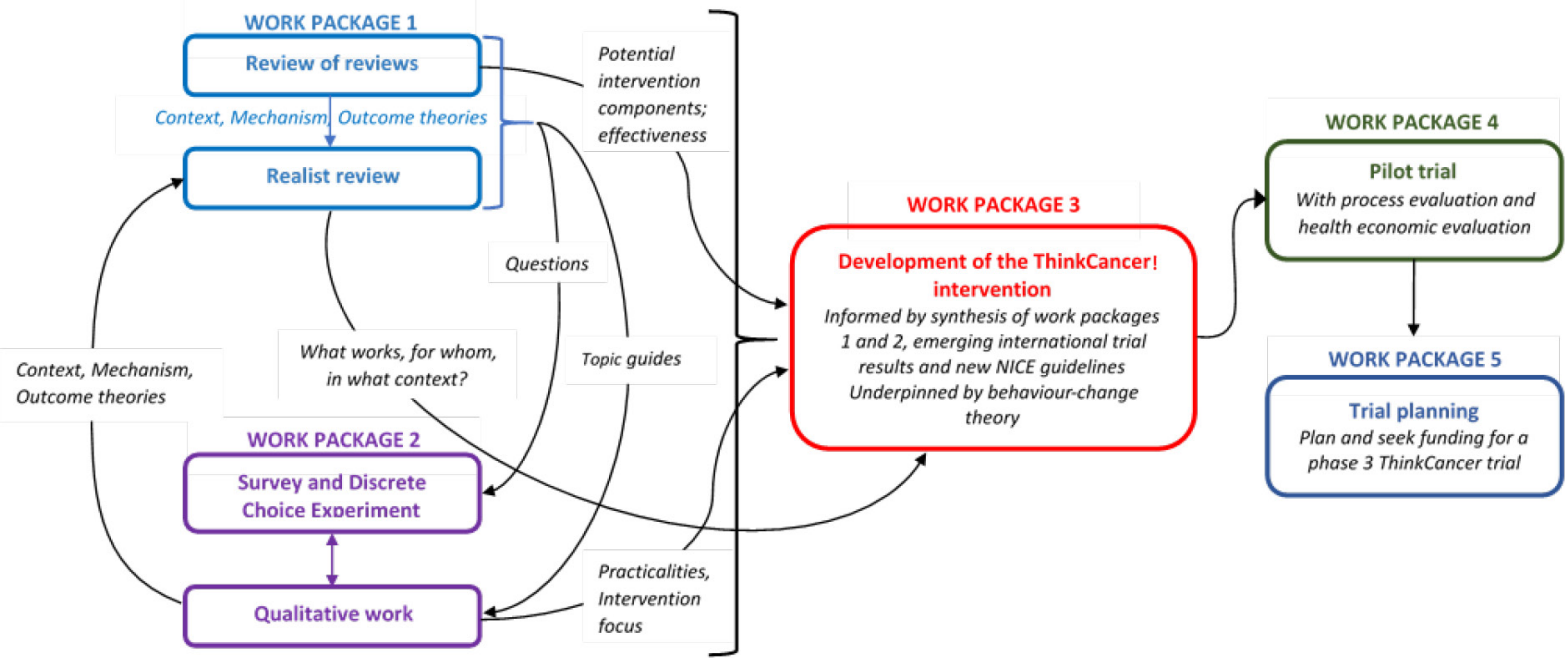
Development of the ‘ThinkCancer!’ intervention for general practices.

**Figure 2.**
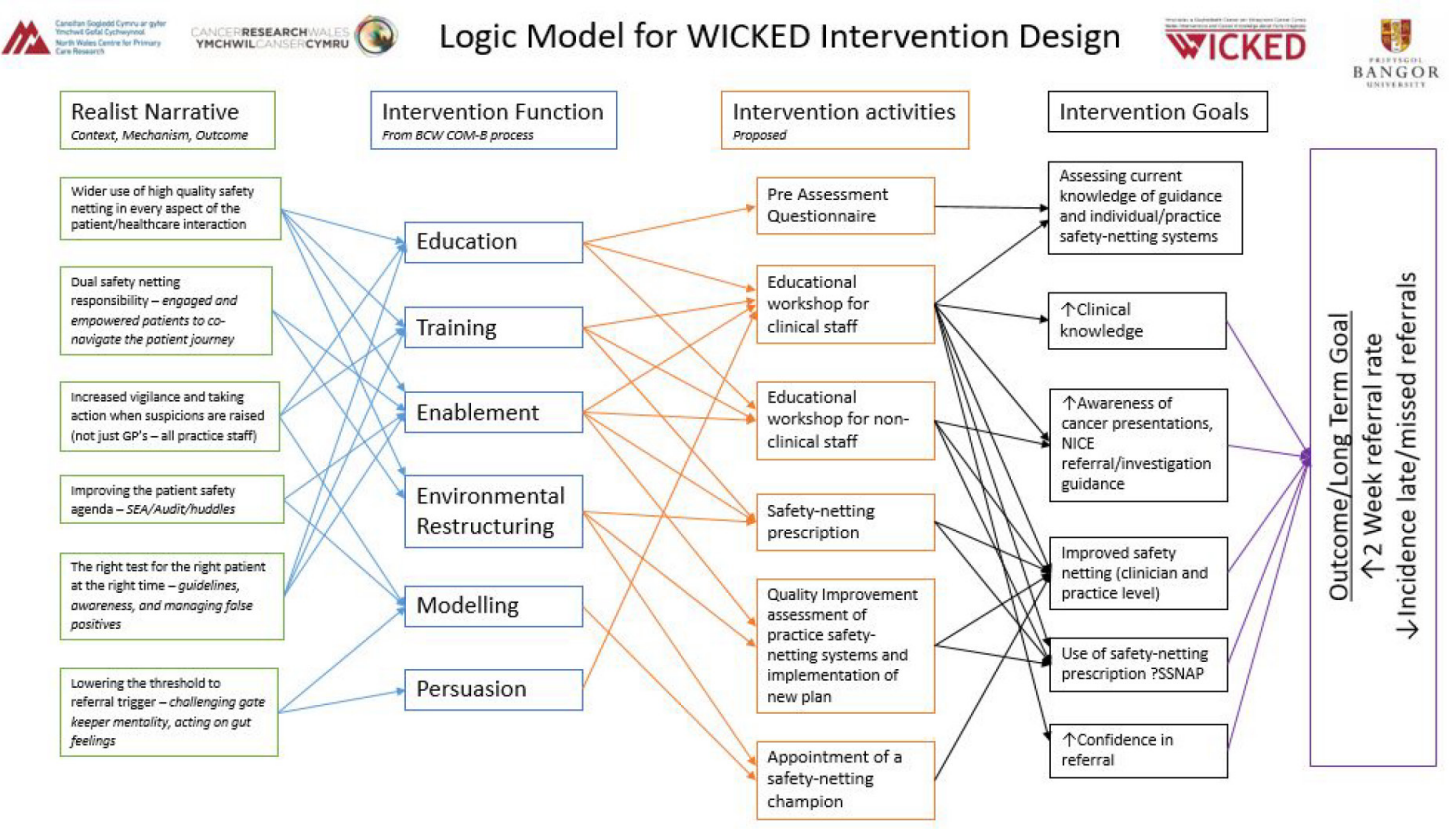
Logic model for design of the intervention.

The development of the intervention ‘ThinkCancer!’ is summarised in Figures 1 and 2.

The Behaviour Change Wheel informed a three-workshop model, adapted to a digital format.^20^ Key aspects include effective safety netting at practitioner and practice system level, increased vigilance, and liberalised referral thresholds. The 'Early Diagnosis' session was aimed at clinical practice staff and consisted of a teaching seminar followed by a discussion on current safety-netting practices. Participants were supplied with a variety of tools including the more detailed 'ThinkCancer!' handbook, external resources such as NICE guidance, and the Shared Safety Net Action Plan (SSNAP) tool.^21^ The 'Cancer Aware' session was aimed at non-clinical staff and comprised an interactive card game to promote cancer awareness and confidence. The 'Safety Netting' session brought the entire team together, with co-production of a bespoke cancer safety-netting plan (CSNP) and appointment of a cancer safety-netting champion.^22^ Paper materials were sent prospectively to practices, and electronic resources were made available.

Primary clinical outcomes related to suspected cancer referral, including the urgent suspected cancer (USC) (or 2-week wait [2WW]) referral rate and the primary care interval (PCI), and were collected at practice level from both healthcare professionals and practice systems. The 2WW referral rate is defined as the annual number of 2WW referrals divided by practice list size, which is then multiplied by 100 000.^15^ The PCI is defined as the time between the date of first presentation and the date of referral.^2^ We did not expect to detect changes to these clinical measures in this feasibility study due to the small sample size and short follow-up duration. Further outcomes tested the feasibility of the intervention and its iterative development and refinement, and informed the design of the Phase III trial, and were measured using an adapted NoMAD survey and through interviews.^23^ The full list of outcome measures and data collection methods can be viewed in more detail in Table 1.

**Table 1.** Outcome measures

Outcome measure	Source	Group	Level	Time points
2WW	CRF	Intervention and control	Practice level	Baseline and 6 months follow up
PCI				
Conversion rate				
Detection rate				
*Feasibility measures*				
Recruitment	Recruitment log	Intervention and control	Practice level	Monitored throughout until end of recruitment period
Retention	Recruitment log	Intervention and control		Monitored throughout until end of follow up
Adherence	Feedback forms, interviews, post-workshop reflections	Intervention		Monitored throughout until end of follow up
Fidelity				Monitored throughout until end of follow up
Data collection	Completed CRF forms returned; completion of feedback forms and NoMAD surveys	Intervention and control	Practice level and individual staff level	End of follow up
*Descriptive measures*				
Practice characteristics	Practice questionnaire, interviews	Intervention and control	Practice level	Baseline and 6 months follow up
	Reflective notes	Intervention		
Existing safety-netting practices	Staff interviews, feeback forms	Intervention and control	Practice level	Baseline and 6 months follow up
*Process evaluation measures*				
Acceptability	Staff interviews, Feedback forms	Intervention	Individual participants	2 months post-workshop
Implementation	NoMAD survey, interviews	Intervention	Individual participants	2 months post-workshop
*Health economic measures*				
Intervention delivery costs	Health economics data collection sheets	Intervention	Intervention deliverers	Immediately following each workshop
Staff attendee time	Intervention deliverer workshop notes	Intervention	Individual participants	Notes recorded during each workshop

2WW = 2-week wait. CRF = Case reporting form. PCI = Primary care interval.

Data collection forms to measure clinical outcomes were completed on paper and entered in MACRO (version 4.9). The practice feedback questionnaires and the NoMAD survey were digitalised to allow remote collection via a SurveyMonkey link.

### Qualitative methods

The qualitative interview guide was amended to incorporate questions on the impact of the pandemic on practices, as it quickly became clear that primary care had been significantly affected. Qualitative telephone interviews were conducted with a sample of 16 practice staff (Supplementary Table S1) from both arms of the study. Interviews lasted 30–60 minutes and were recorded, fully transcribed, and analysed using Framework, a 5-stage matrix-based method for analysing qualitative data.^24^

### Health economic methods

From an NHS perspective, micro-costing methodology was used to assess the feasibility of gathering sufficient economic data to cost the intervention delivery. Cost information was collected via data collection sheets completed by intervention deliverers. Budget impact analysis and sensitivity analysis were conducted following our base-case costings to assess the potential costs of face-to-face delivery as initially planned before the COVID-19 pandemic. A full description of the health economic methods is available elsewhere.^25^

## Results

**Figure 3.**
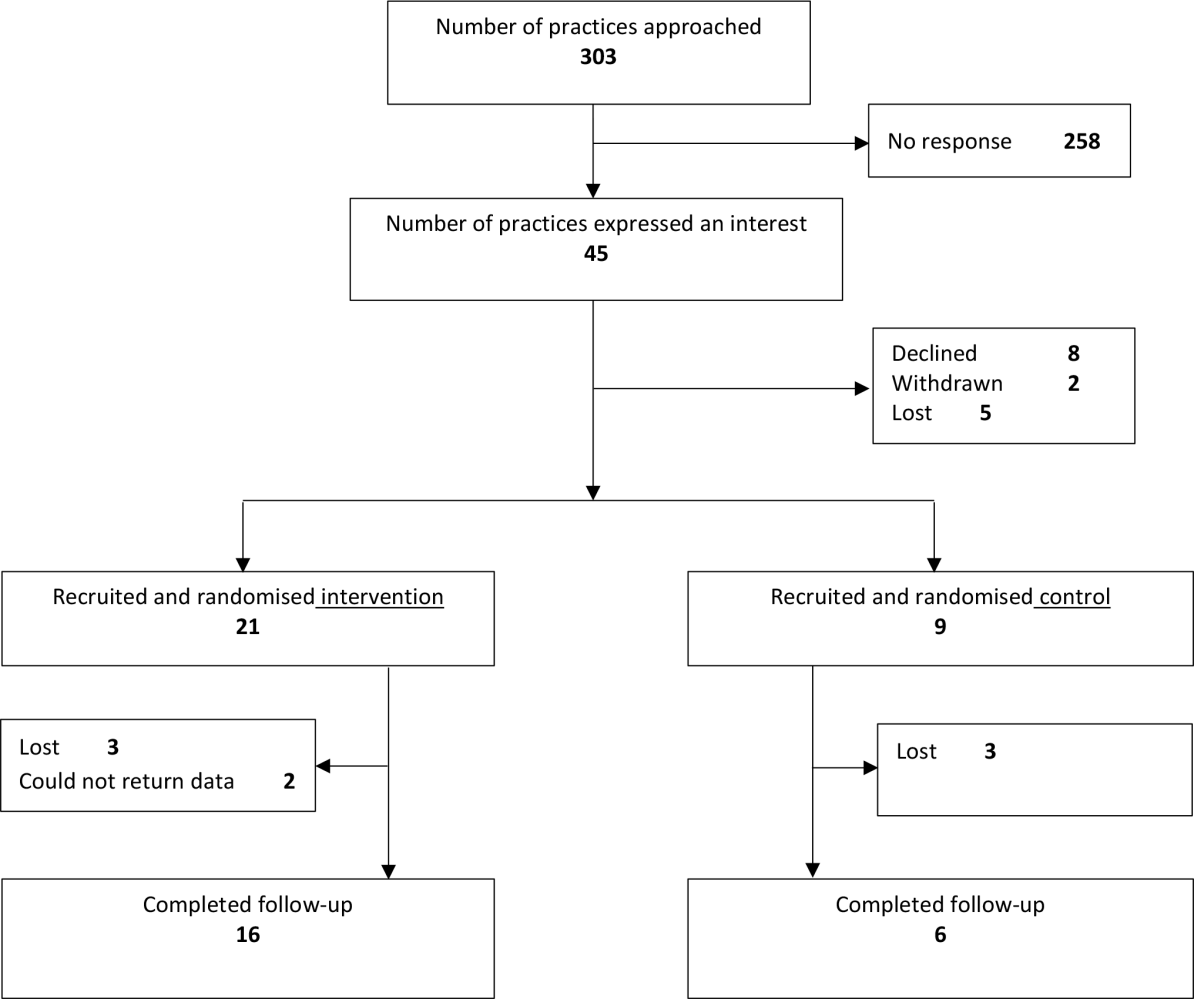
CONSORT diagram of study participant (general practices) flow.

We approached 303 practices across all seven health boards in Wales(Figure 3). Of these practices, 45 expressed interest and eight declined. Following expression of interest, two practices withdrew and five were lost to the trial. The remaining 30 practices were randomised: 21 to intervention and nine to control. Following randomisation, six practices were lost, mainly due to the impact of the pandemic. Two practices were still in contact at the end of the data collection period but were unable to return follow-up data by the deadline for locking the trial databases. There were 22 practices who returned some of the follow-up data. No harms were recorded.

Results of the progression criteria are presented in Table 2. Recruitment, intervention fidelity, and obtaining routine data were in the GO category (successful for a definitive trial). Retention, staff-level fidelity, and ability to collect individual level data were in the review category; improvement strategies were designed. Practice questionnaire data is presented in Supplementary Table S2.

**Table 2.** Progression criteria results

Criterion	Stop	Review	Go	Result
Confirmation of adequate recruitment for a definitive trial at practice level	<15 practices recruited	15–19 recruited	≥20 general medical practices recruited	30/30 recruited (100%) GO
Confirmation of adequate retention for the definitive trial at practice level	<65% practices retained	65–79% practices retained	≥80% practices retained	22/30 remained in study at follow up (73%) REVIEW
Confirmation of adequate fidelity of the intervention	<50% of interventions delivered	50–79% of all interventions delivered	≥80% of all intervention sessions delivered	19/21 interventions delivered (90%) GO
Confirmation of adequate fidelity at individual practice staff level	≥50% of the clinical staff per practice should attend the workshops;			*Session 1:* 6/19 (32%) *Session 3:* 4/19 (21%) REVIEW
	≥50% of the administrative staff per practice should attend the workshops, comprising ≥50% of the reception and secretarial staff as well as the practice manager.			*Administrative* Session 2: 7/19 (37%) Session 3 – 4/19 (21%) REVIEW
				*Reception* Session 2: 2/19 (11%) Session 3: 0/19 (0%) REVIEW
	≥75% of the staff should receive the training either directly or indirectly.^a^			Session 1: 3/9 (33%) Session 2: 3/9 (33%) Session 3: 3/9 (33%) REVIEW
*Progression criteria relating to obtaining data regarding completion of outcome measures was be assessed using the following criteria:*				
Obtaining routine data		Data from <70% of practices obtained	Data from ≥70% practices obtained	22/30 returned follow up data (73%) GO
Obtaining individual data		Data from <70% of individuals from each practice obtained	Data from ≥70% of individuals of each practice obtained	*Workshop evaluation form* Completed by 67 participants <70% *Adapted NoMAD* Completed by 39 participants <70% REVIEW

^a^ Data only available from 9 practices.

Safety netting was assessed using quantitative and qualitative measures (Supplementary Table S3, Figure S1). Most (79%) of practices kept a register of USC referral; repeat consultations were highlighted in just under half. Most did not routinely check USC appointments attendance or whether investigations had been done, nor book routine follow-ups. Safety-netting responsibilities did not go beyond clinical staff, there was regular communication regarding cancer patients among clinical staff, but not among staff generally.

The safety-netting plans designed by 19 intervention practices were examined (two were unable to complete all workshops). Four identified the need for a formal USC referral register. Twelve planned to formalise their safety netting using the Cancer Research UK/Macmillan information sheets (CRUK 2020). Seven planned to enhance their ‘did not attend’ follow-up practices, and 11 to formally establish a 4–6-week follow up for all those referred. Ten planned to enhance their use of significant event audits for USC referrals and ten planned to undertake more advanced or bespoke audit projects. More advanced safety-netting measures were proposed by many of the practices; these included continuity of care actions, challenges to ‘downgrading’ of 2WW referrals by secondary care, and challenges to failed or delayed investigations and appointments resulting from 2WW referrals.

Results from the outcome measures are presented in Table 3. Details of the analysis models applied to the data are in the table. Data analysis was performed using Stata 16 and R4.0.2. Data were queried from practices, if necessary; data cleaning was completed by the trial statistician; and an independent Trial Steering Committee oversaw the trial, including data quality.

**Table 3.** Analysis model combining PCI, conversion, and detection rates data.

Outcome measure	Source	Descriptive	Control baseline	Control follow up	Intervention baseline	Intervention follow up
PCI	Raw data	*n*	53 patients, 6 practices	190 patients, 6 practices	235 patients, 18 practices	159 patients, 15 practices
		Mean (SD)	8.5 (22.7)	14.0 (37.4)	15.4 (47.6)	25.6 (94.9)
		Median [IQR]	0 [0 4]	1 [0 10]	1 [0 11]	2 [0 22]
		Minimum, maximum	0, 128	0, 373	0, 582	0, 1106
	Analysis model	Multilevel Mixed Effects Generalised Linear Model with cancer type and health board as factors, allocated group, time, and a time*group interaction with GP practice as a random effect, with a negative binomial distribution and log link function				
		Adjusted mean	4.6	19.3	15.0	17.8
2WW referral rate	Raw data	*n*	6	6	18	16
		Mean (SD)	1,158 (730.6)	1710.7 (626.3)	1538.5 (516.5)	1636.8 (806.4)
		Median [IQR]	991 [789 1959]	1571.5 [1514 1,724]	1364.5 [1226 2018]	1386.5 [1189.5 1986]
		Minimum, maximum	154, 2064	999, 2884	770, 2343	380, 3364
	Analysis model	Analysis of covariance with follow-up results as the dependent variable, baseline result included as a covariate, and health board and allocated group as factors				
		Adjusted mean		1882.5		1572.4
Conversion rate	Raw data	*n*	6	6	18	15
		Mean (SD)	0.11 (0.04)	0.11 (0.05)	0.14 (0.06)	0.22 (0.22)
		Median [IQR]	0.11 [0.07 0.11]	0.12 [0.08 0.15]	0.14 [0.09 0.17]	0.18 [0.13 0.19]
		Minimum, maximum	0.07, 0.18	0.03, 0.15	0, 0.26	0.06, 1
	Analysis model	Fractional response regression, with follow-up value as dependent variable, the baseline value as covariate, and allocated group and health board as factors				
		Adjusted mean		0.12		0.22
Detection rate	Raw data	*n*	5	6	18	15
		Mean (SD)	0.95 (0.11)	0.94 (0.13)	0.73 (0.26)	0.72 (0.25)
		Median [IQR]	1 [1 1]	1 [0.98 1]	0.81 [0.45 0.93]	0.75 [0.64 1]
		Minimum, maximum	0.75, 1	0.67, 1	0.23, 1	0.10, 1
	Analysis model	Fractional response regression, with follow-up value as dependent variable, the baseline value as covariate, and allocated group and health board as factors				
		Adjusted mean		0.96		0.74

IQR = Interquartile range. PCI = Primary care interval. SD = standard deviation.

For the PCI, the adjusted mean at follow up was 19.3 for the control group and 17.8 for the intervention group, and for the 2WW referral rate the adjusted mean at follow up was 1882.5 for the control group and 1572.4 for the intervention group. The conversion rate (CR) is the proportion of 2WW referrals that could be cancer. The adjusted mean at follow up was 0.12 for the control group and 0.22 for the intervention group. The detection rate (DR) is the proportion of patients with a diagnosis of cancer who had a 2WW referral. The adjusted mean at follow up was 0.96 for the control group and 0.74 for the intervention group.

### Process evaluation and qualitative work

Results of the adapted NoMAD are presented in Supplementary Table 4. The NoMAD was completed by fewer staff than hoped. Some staff are integrating the CSNP into their work, however some clinical and non-clinical staff need some further persuasion.

Interviews with practice staff were conducted to explore the acceptability of the intervention, safety netting, data collection, intervention uptake, SSNAP tool and implications for staff, practice, and patients. Overall, the intervention was viewed in a positive light and was well-received. The practice questionnaire was deemed straightforward; however, collection of the PCI and USC data were time consuming. Remote workshop delivery was successful, allowing more staff members to attend. Pre-recorded workshop sessions were found useful. The interactive elements of the workshops and the whole-practice approach were valued. Delivery by a GP educator was considered an asset. The creation of the CSNP was useful for knowledge-sharing. All intervention practices created a CSNP and appointed a cancer safety-netting champion. The intervention resources such as the handbook were seen as useful, except SSNAP tool as it was not an e-resource.

Interview participants felt the ‘ThinkCancer!’ intervention worked to revive safety-netting systems and implement changes. Non-clinical participants had raised awareness of potential cancer symptoms, increased confidence, and a sense of reassurance.

### Health economics

Data collection methods were feasible to collect sufficient health economics data to cost the delivery of ‘ThinkCancer!’. The total cost of delivering ‘ThinkCancer!’ to 19 general practices was £25 030 accounting for intervention delivery time, staff attendee time, and materials. The average cost per practice was £1317. Findings from the budget impact analysis indicated a total of £34 630 for face-to-face delivery based on additional travel time and mileage costs.^25^

## Discussion

### Summary

The rigorously developed and novel ‘ThinkCancer!’ intervention proved feasible, acceptable, and welcomed by practices across Wales. Existing practice (for example referral registers and safety netting) was varied, illustrating room for improvement. Safety-netting practices often met the CRUK and Macmillan guideline criteria, yet practices were keen to make further improvements.

### Strengths and limitations

A key strength was that a definitive trial was recommended after the successful progression criteria for recruitment, intervention fidelity, and obtaining routine data. Agile, iterative adaptation of the intervention continued throughout, addressing individual practice needs. The systemic, whole-practice approach used by ‘ThinkCancer!’ is novel and was endorsed by both clinical and non-clinical staff.

All practices created bespoke CSNPs, with many concurrent actions across the practices, such as serious adverse events scrutiny, audits, and formalised safety-netting approaches and follow up.

Remote delivery proved highly advantageous and improved practice recruitment. In Phase III, multiple workshops could be delivered across multiple practices without the need for travel. Caution will be needed assessing dose and reach. Registration systems will be built into future online workshop sessions to better capture attendance.

Weaknesses were that retention, staff-level fidelity, and individual-level data collection needed improvement. These are mitigated by new strategies for the Phase III trial. Accuracy of self-reported clinical data obtained from practices was less than anticipated; research officer collection will replace this. We hope to improve capture of the PCI, USC rate, CR, and DR data, and to lower primary care clinician burden.

For reasons explained here, the self-reported data the PCI, 2WW, CR, and DR do not reflect what would have been expected based on previously published data. The sample size was too small to make inferences regarding intervention impact; rather this feasibility work aimed to see if the data collection was possible and to test analysis models. For the PCI, identification of the first symptom or sign suggesting cancer was difficult for practice staff. Ideally the PCI should reduce in the intervention group from baseline to follow up. In this small sample, the raw USC referral rate data were lower than those prior to the COVID-19 pandemic, and adjusted USC referral rate was higher in the control compared to intervention group. The adjusted conversion rate was higher in the intervention than control group. Both were counterintuitive findings if the threshold for referral has been lowered.

Another limitation was delay between randomisation and workshop delivery. Intervention fidelity indicated feasibility; more than 80% of interventions booked were delivered, but specified time limits will be set for a future trial. Workshop attendance and dissemination were hard to measure as was the implementation of safety-netting plans; this has been modified for a main trial.

### Comparison with existing literature

To the best of our knowledge, this trial is unique, as no other trials are examining interventions aimed at the whole general practice team. One Australian trial addressing primary care and improving cancer diagnosis differed substantially as it only addressed one item of the six from our programme theory.^26^ The ongoing UK trial called CasNet concentrates mostly on safety netting.^27^

### Implications for research and practice

The improvement in cancer diagnosis over time due to increased 2WW referrals, and other modelling studies, suggest that referral thresholds in primary care should potentially be liberalised further.^28^ However, the pressure on secondary care from these referrals and acute lack of access to diagnostic testing, which is far greater in other countries, makes this a difficult job in primary care.^29^ The spread of rapid diagnostic clinics in the UK is designed to partially overcome the diagnostic testing gap, but more direct access is also needed in general for primary care. Interventions such as ‘ThinkCancer!’ should empower primary care to make scientifically sound referrals and bring pressure to bear for faster access to diagnostic tests and facilities. We previously reported the cost of the ‘ThinkCancer!’ intervention and recognise that this can only be implemented (if effective) in healthcare systems with comprehensive primary care.^25^ However, the principles of both timely referral and safety netting will apply in any health system.

Informed by the results of this feasibility work and other literature, a sample size for a definitive RCT has been calculated based on the PCI as the primary outcome, analysed using time to event models. Assuming a median PCI of five in the intervention group and four in the control group, it will be necessary to recruit 76 practices to achieve 90% power at a 5% significance level, based on an average of 53 patients per practice per year having a diagnosis of cancer.

Key areas for future research (other than the Phase III trial now in progress) include comparisons of measurement of the guideline interval versus the primary care interval, cancer type-specific future interventions for primary care settings, and comparisons of safety-netting interventions, particularly the balance between the role of the patient and the role of primary care.

Key stakeholders from the inception of the study included policy makers, practice staff, Patient and Public Involvement (PPI) contributors, patient forums, and patients and carers. This project has positively influenced cancer policy in Wales; success was also illustrated by recent funding of the Phase III trial by Cancer Research Wales and Northwest Cancer Research. With the increased focus on the early diagnosis of cancer across Wales, ‘ThinkCancer!’ could potentially play a central role.

## Data

The dataset relied on in this article is available from the corresponding author on reasonable request.
